# Healthcare demand in response to rabies elimination campaigns in Latin America

**DOI:** 10.1371/journal.pntd.0007630

**Published:** 2019-09-26

**Authors:** Jonathan Yoder, Elisabeth Younce, Felix Lankester, Guy H. Palmer

**Affiliations:** 1 School of Economic Sciences, Washington State University, Pullman, Washington, United States of America; 2 Paul G. Allen School for Global Animal Health, Washington State University, Pullman, Washington, United States of America; Universidad Nacional Mayor de San Marcos, PERU

## Abstract

The World Health Organization, the World Organization for Animal Health, and the Food and Agriculture Organization have resolved to eliminate human rabies deaths due to dog bites by 2030, and the Vaccine Alliance (Gavi) has added human rabies vaccines to their investments for 2021–2025. Implementing these goals cost-effectively and sustainably requires understanding the complex connections between dog rabies vaccination and human risk and response. The objective of this paper is to estimate how dog rabies vaccinations affect human rabies deaths, mediated through dog rabies cases, dog bite reporting, and post-exposure human rabies vaccination. To approach this objective, we apply multivariate regression analysis over five rabies-related outcomes: (a) dog vaccinations, (b) dog rabies cases, (c) reported human exposures, (d) human post-exposure prophylaxis (PEP) use, and (e) human rabies cases. Analysis uses aggregate annual data over 1995–2005 for seven Latin American countries that experienced dramatic declines in canine and human rabies. Among other results, we estimate the following. (i) A 10% increase in dog vaccinations decreases dog rabies cases by 2.3%. (ii) Reported exposures decline as concurrent dog rabies cases decline, but these declines are more than offset by increases in reported exposures per dog rabies case, which may result from higher rabies awareness due to anti-rabies campaigns. (iii) A 10% increase in PEP use decreases human deaths by 7%, but a 10% increase in dog vaccination induces a 2.8% decrease in PEP use. The net effect is that a 10% increase in dog vaccination reduces human deaths by 12.4% overall, although marginal effectiveness declines as dog rabies incidence declines. (iv) Increases in income and public health expenditures increase PEP demand. The findings highlight the importance of mass dog vaccination, heightened awareness, treatment access, and clinical algorithms to reduce both false negatives leading to death and false positives leading to costly unnecessary PEP prescriptions.

## Author summary

Several global health organizations have prioritized investment for global elimination of human death from canine rabies. Cost-effective deployment requires understanding complex connections between rabies risk and healthcare seeking behavior. During 1995–2005, there was a rapid decline in dog and human rabies cases in several Latin American countries following concerted investment in mass dog vaccinations and human post-exposure vaccination. Using data from this period, we econometrically estimate relationships between dog vaccinations, dog rabies cases, human exposure reports, human post-exposure prophylaxis (PEP) use, and human rabies deaths. We estimate quantitatively how dog vaccinations indirectly reduce human rabies deaths, mediated through dog rabies cases, dog bite reporting, and PEP use. We find that reported human exposures decline as dog rabies cases decline, but these declines are offset by increases in reported exposures per dog rabies case, which may be driven by increased rabies awareness through anti-rabies campaigns. Furthermore, while PEP demand declines as dog rabies cases decline, increases in income and public health expenditures concurrently increase demand for PEP. These findings provide better understanding of the underlying factors driving PEP demand and exposure (under)reporting to inform cost-effective policy design to reduce unnecessary PEP prescription and false negatives leading to human death.

## Introduction

In November 2018, The Vaccine Alliance (Gavi) added human rabies vaccines to investments for 2021–2025 [1]. This investment in human vaccine for post-exposure prophylaxis aligns Gavi with the tripartite goal of the World Health Organization, World Animal Health Organization, and Food and Agriculture Organization to eliminate rabies as a cause of human death [2]. While rabies was ranked high for its public health impact in Gavi’s analysis for vaccine prioritization, concern was raised regarding the operational complexity of human post-exposure prophylaxis (PEP) as cost effective control requires progressive reduction in the zoonotic transmission of rabies and thus decreased demand for PEP over time. Given that bites from rabid dogs are responsible for over 95% of the annual 59,000 human deaths [3, 4], sustainable elimination of human deaths will likely require both Mass Dog Vaccination (MDV) and improved access to PEP for bite victims [5, 6]. With sufficient coverage, MDV is effective in eliminating transmission source and is significantly more cost effective than relying solely on PEP [7,8]. Nonetheless, access to PEP is necessary following bites of unvaccinated dogs or dogs for which the vaccination and disease status is uncertain [9,10].

Understanding the budgetary commitment required for eliminating and maintaining elimination on a national and regional scale is essential, especially given that the remaining burden of dog mediated rabies falls exclusively on low- and middle-income countries (LMIC) [4]. Reliance on PEP alone represents a continuous cost burden [3,8]. In contrast, when PEP is combined with MDV reaching consistent 70% coverage, models based on studies in sub-Saharan Africa suggest that the total cost per averted death would decrease relatively rapidly [8]. The most compelling evidence for this has been witnessed in eight Latin American countries where national rabies control programs initiated a regional elimination effort in 1983 incorporating both MDV and improved access to and subsidization of PEP [12,13]. This effort, although still short of complete elimination [11, 14–16], resulted in a sustained 95% reduction in human rabies deaths by 2012 [11, 14–16]. However, the evidence from these sustained national campaigns illustrates not only the effectiveness of MDV, but also suggests a cautionary note: While the number of rabid dogs dramatically decreased, consistent with the efficacy of MDV, the number of doses of PEP has increased in recent years [16].

The objective of this paper is to quantitatively estimate the relationships among dog vaccinations and human rabies deaths, mediated through dog rabies cases, while controlling for dog bite reporting and PEP use. To do so, we use an integrated econometric model of the relationships among i) dog vaccinations, ii) dog rabies cases, iii) suspected human exposure; iv) PEP use, and v) human rabies cases. The model accounts for intertemporal dynamics that relate current and past vaccinations and dog rabies cases to be propagated over time, and accounts for PEP demand, reflecting both the perceived risk of acquiring rabies given an exposure, and the reduction in its likelihood. The model allows estimation of an extensive set of of indirect and conditional epidemiological relationships and human decisions that drive the connection between dog vaccinations and human rabies death.

## Data and methods

**Fig 1:**
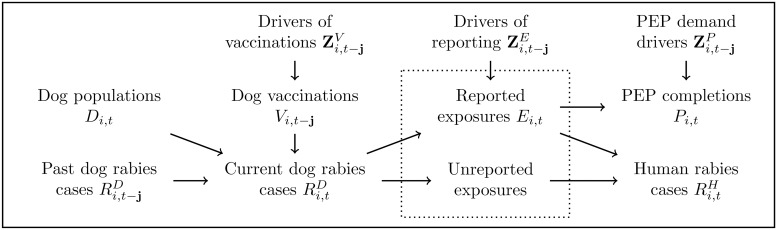
Visual relationship among system elements.

Fig 1 illustrates key elements of the system. Dog Rabies Cases occur within a larger Dog Population *D*_*i*,*t*_ [17]. Current Dog Rabies Cases $\left(R_{i,t}^{D}\right)$ in country *i*, year *t* depend in part on the number of past Dog Rabies Cases $\left(R_{i,t-j}^{D}\right)$, where **j** ∈ (0,1,…) represents a general lag structure. Dog Vaccinations (*V*_*i*,*t*−j_) reduces susceptibility and affects current Dog Rabies Cases [18]. Vaccination efficacy may last multiple periods, so previous years’ vaccinations may affect current outcomes. Dog vaccination decisions occur at the intersection of supply policy decisions (i.e. MDV) and willingness of households to participate [14]. Factors affecting vaccination rates are represented by $Z_{i,t-j}^{V}$, (bold font represents a set of variables).

Reported [human] Exposures (*E*_*i*,*t*_) represent reports of contact with animals that may be (but are not necessarily) infected with rabies. Contact victims may or may not report a bite that would have been identified clinically as an exposure [19]. Drivers of reporting $\left(Z_{i,t-j}^{E}\right)$ include information, education, past experience with rabies or rabies vaccination campaigns, income, and other factors that may affect the reporting rate. The underreporting rate affects the number of unreported exposures, which are unobserved and absent from our data [20]. Current Reported Exposures may be treated with a course of PEP, contributing to the number of PEP Completions (*P*_*i*,*t*_).

If PEP is not acquired by an exposed and infected victim, that individual will contribute to the number of Human Rabies Cases $\left(R_{i,t}^{H}\right)$, which invariably leads to death [21]. Reported exposures that receive timely PEP [course] Completions do not develop rabies symptoms, do not die of rabies, and therefore are not counted among Human Rabies Cases. The decision to receive PEP treatment given exposure reporting is affected by PEP demand drivers $Z_{i,t-j}^{P}$, including infection risk as perceived by the victim, their health care providers and informal advisors, income, out-of-pocket PEP acquisition cost, and potentially other factors [19,22]. While not all of these variables are available, we accounted for each of these broad categories of variables as well as possible given available data, in some cases using proxy variables. Additional theoretical background is presented in S1 File.

### Data and sources

Our data represent seven countries (Brazil, Colombia, Peru, Venezuela, Nicaragua, Dominican Republic, Mexico), which were substantially affected Latin American countries between 1995 and 2005, and for which we have the most reliable data. The data were compiled from a series of reports published by the Reunión de Directores de los Programas de Rabia de las Américas (REDIPRA) from 1995–2006, based on surveys implemented by the Pan American Health Organization in tandem with semiannual REDIPRA meetings [13]. Table 1 summarizes data descriptions and sources, and Table 2 provides summary statistics. The data span 1995 through 2005 for seven countries, for a potential sample frame of N(max) = 77. However, dog population estimates are missing for 1995 and 1996, and vaccination data are missing for 1995–1997. Further, regression sample sizes are also limited because of the use of lagged values in the regressions. Table 3 includes summary statistics for all observations for each variable that are used in any of the regressions reported in results.

**Table 1:** Data descriptions of variables used in analysis.

Variable		Description
Dog Vaccinations	*V*_*i*,*t*_	Number of dogs vaccinated against rabies (1,000s). (REDIPRA 1992–2006). [13]
Dog Rabies Cases	$R_{i,t}^{D}$	Number of reported confirmed dog rabies cases (REDIPRA 1992–2006). [13]
Reported Exposures	$E_{i,t}^{H}$	Number of people reported to have had physical contact with a suspected rabid animal (REDIPRA 1992–2006). [13]
PEP Completions	*P*_*i*,*t*_	Number of people who completed a full course of PEP (REDIPRA 1992–2006). [13]
Human Rabies Cases	$R_{i,t}^{H}$	Number of reported human rabies cases. Based on REDIPRA reports (REDIPRA 1992–2006), corroborated with the SIRVERA database (http://sirvera.panaftosa.org.br). Human cases can be \interpreted as human deaths. [13]
Dog Population	*D*_*i*,*t*_	Dog population. Estimation methods vary per country (thousands). Imputation for missing data used for this paper is described in the text (REDIPRA 1992–2006). (1,000s). [13]
Urban Population	$H_{i,t}^{u}$	[Human Population, total] times [Urban population (% of total)]/(1,000). World Bank indicator codes: SP.POP.TOTL and SP.URB.TOTL.IN.ZS, respectively. (1,000s). [23]
Rural Population	$H_{i,t}^{r}$	$\left(H_{i,t}-H_{i,t}^{urb}\right)$, where H_i,t_ is Human Population, total, World Bank indicator codes: SP.POP.TOTL. (1,000s). [23]
Income	*I*_*i*,*t*_	GDP per Capita, PPP, constant 2011 international $. World Bank Indicator Code NY.GDP.PCAP.PP.KD. [23]
Health Expenditures	*X*_*i*,*t*_	Health expenditure per capita, PPP constant 2011 international $. World Bank Indicator Code SH.XPD.PCAP.PP.KD. [23]
Out of Pocket	*O*_*i*,*t*_	[X_i,t_] times [Out-of-pocket health expenditure (% of total expenditure on health)]. World Bank indicator code SH.XPD.OOPC.TO.ZS. [23]
BMP	*BMP*	An indicator variable representing Brazil, Mexico, and Peru used in the Human Rabies cases regression (Table 6). Explanation provided in Supplementary Materials.

**Table 2:** Descriptive statistics.

Variable		N^a^	mean	median	sd	min	max
Dog Vaccinations	(*V*_*i*,*t*_)	53	5,314	1,733	6,706	36	18,514
Dog Rabies Cases	$\left(R_{i,t}^{D}\right)$	67	187	76	314	1	1,746
Reported Exposures	(*E*_*i*,*t*_)	67	101,378	53,055	131,025	10,159	466,224
PEP Completions	(*P*_*i*,*t*_)	67	38,631	5,289	79,743	549	268,326
Human Rabies Cases	$\left(R_{i,t}^{H}\right)$	67	5.87	2	8.08	0	28
Dog Population	(*D*_*i*,*t*_)	60	6,934	3,197	7,187	523	21,771
Urban Population	$\left(H_{i,t}^{u}\right)$	67	44,331	22,471	47,571	2,528	154,831
Rural Population	$\left(H_{i,t}^{r}\right)$	67	12,697	6,996	11,628	2,173	35,783
Income	(*I*_*i*,*t*_)	67	9,810	8,634	3,721	3,076	15,619
Health Expenditures	(*X*_*i*,*t*_)	67	433	425	178	126	899
Out of Pocket	(*O*_*i*,*t*_)	67	180	171	97	35	401

^a^Statistics in this table represent the sample used in Regressions (3) and (4) reported in Table 3, which have the largest sample sizes.

**Table 3:** Regression results for dog rabies cases, reported exposures, PEP completions, and human rabies cases, respectively.

Regression 1		Regression 2		Regression 3		Regression 4	
Dep. Var: $\text{ln}\;\left(R_{i,t}^{D}\right)$^a^		Dep. Var: ln(*E*_*i*,*t*_)		Dep. Var: ln(*P*_*i*,*t*_)		Dep. Var: $R_{i,t}^{H}$	
Var.	Coef.	Var.	Coef.	Var.	Coef.	Var.	Coef.
$\text{ln}\;\left(R_{t-1}^{D}\right)$	0.56***	ln(*E*_*t*−1_)	0.07	ln(*E*_*t*_)	0.31	$\hat{ln(P_{it})\;}$	-0.71***
ln(*V*_*t*_)	-0.23	ln(*E*_*t*−2_)	-0.86***	$\text{ln}\;\left(R_{t}^{D}\right)$	-0.01	$\text{ln}\;\left(R_{t}^{D}\right)$	0.63***
ln(*V*_*t*−1_)	-0.48	$\text{ln}\;\left(R_{t}^{D}\right)$	0.10***	$\text{ln}\;\left(R_{t-1}^{D}\right)$	0.13**	ln(*E*_*t*_)	-0.27
ln(*V*_*t*−2_)	-0.31**	$\text{ln}\;\left(R_{t-1}^{D}\right)$	0.09***	$\text{ln}\;\left(R_{t-1}^{H}\right)$	0.03**	$\text{ln}\;\left(H_{t}^{u}\right)$	-0.36
ln(*D*_*t*_)	-0.83	$\text{ln}\;\left(R_{t-1}^{H}\right)$	0.02*	$\text{ln}\;\left(H_{t}^{u}\right)$	-2.87**	$\text{ln}\;\left(H_{t}^{r}\right)$	-0.21
ln(*D*_*t*−1_)	-1.73**	ln(*t*)	1.28***	$\text{ln}\;\left(H_{t}^{r}\right)$	1.02	_*BMP*_	1.36***
_Const._	30.1***	ln(*D*_*t*_)	-0.02	ln(*I*_*t*_)	2.05***	_Const._	12.3***
		$\text{ln}\;\left(H_{t}^{u}\right)$	-5.86***	ln(*X*_*t*_)	1.72***	*α*^c^	0.05**
		$\text{ln}\;\left(H_{t}^{r}\right)$	-1.10	ln(*O*_*t*_)	-0.81***		
		Const.	85.4***	Const.	-0.28		
N = 60^b^, χ^2^ = 6597		N = 39, χ^2^ = 99.1		N = 67, *R*^*2*^ = 0.59		N = 67, *R*^*2*^ = 0.27	

***p<0.01,

**p<0.05,

*p<0.1

^a^*R*^*D*^ = Dog Rabies Cases, *V* = Dog Vaccinations, *D* = Dog Population, *E* = Reported Exposures, *R*^*H*^ = Human Rabies Cases, *H*^*u*^ = Urban Population, *H*^*r*^ = Rural Population, *P* = PEP Completions, *I* = [per capita] Income, *X* = Health Expenditures, *O* = Out of Pocket [health expenditures], and *BMP* = indicator variable for Brazil, Mexico, Peru. See Table 1 for descriptions.

^b^Reported sample sizes are different from summary statistics in Table 2 because of missing values for some variables, and because the use of lagged variables decreases the number of observations available for the dependent variable in initial time periods.

^c^Parameter *α* is the dispersion parameter for the Negative Binomial regression.

**Fig 2:**
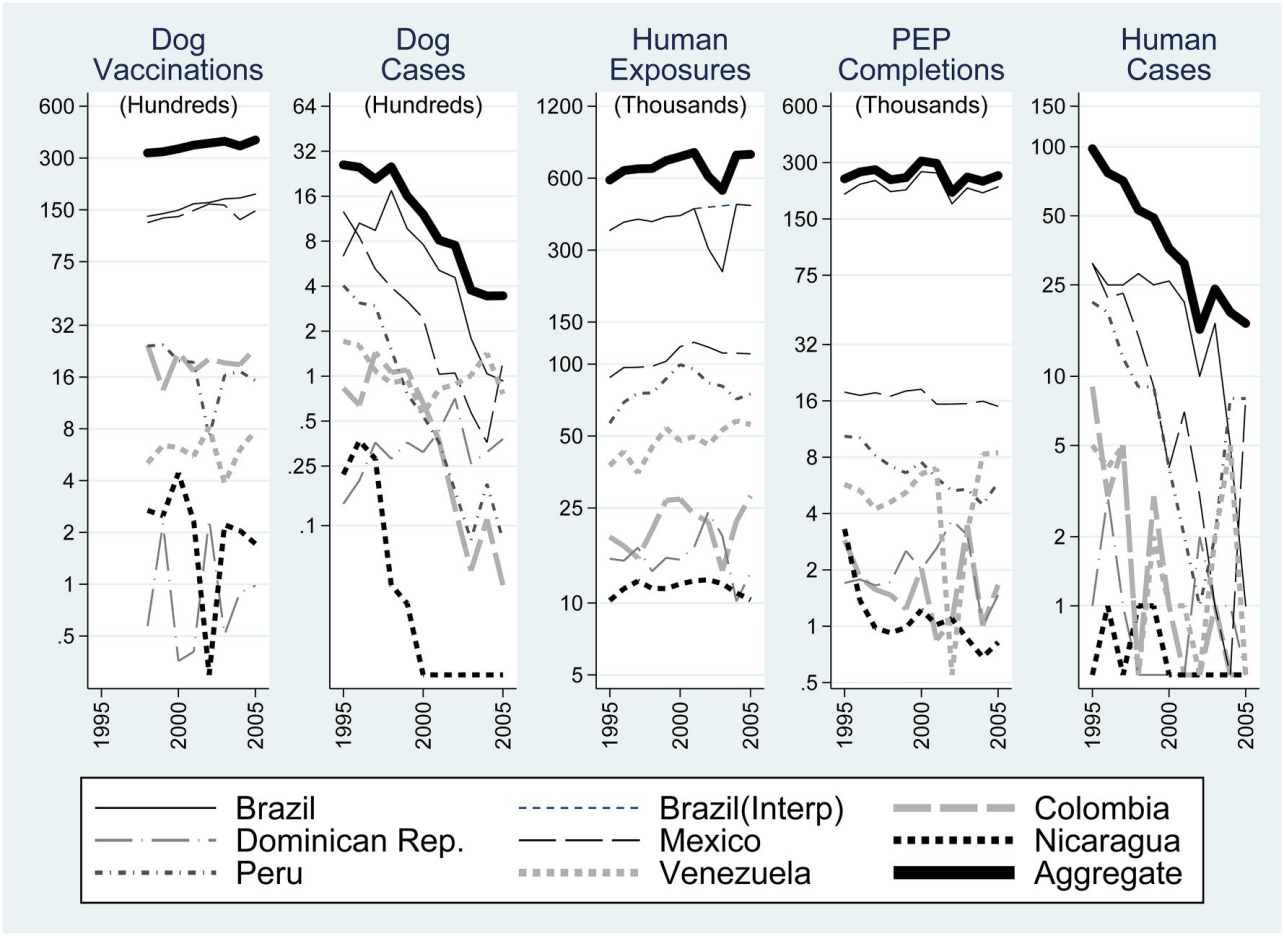
Time series of the five focal variables for each of the seven countries, and the sum across countries. Natural log scale. “Brazil (Interp)” interpolates over a data anomaly in *Reported Human Exposures* discussed in the data section of supplemental materials.

**Fig 3:**
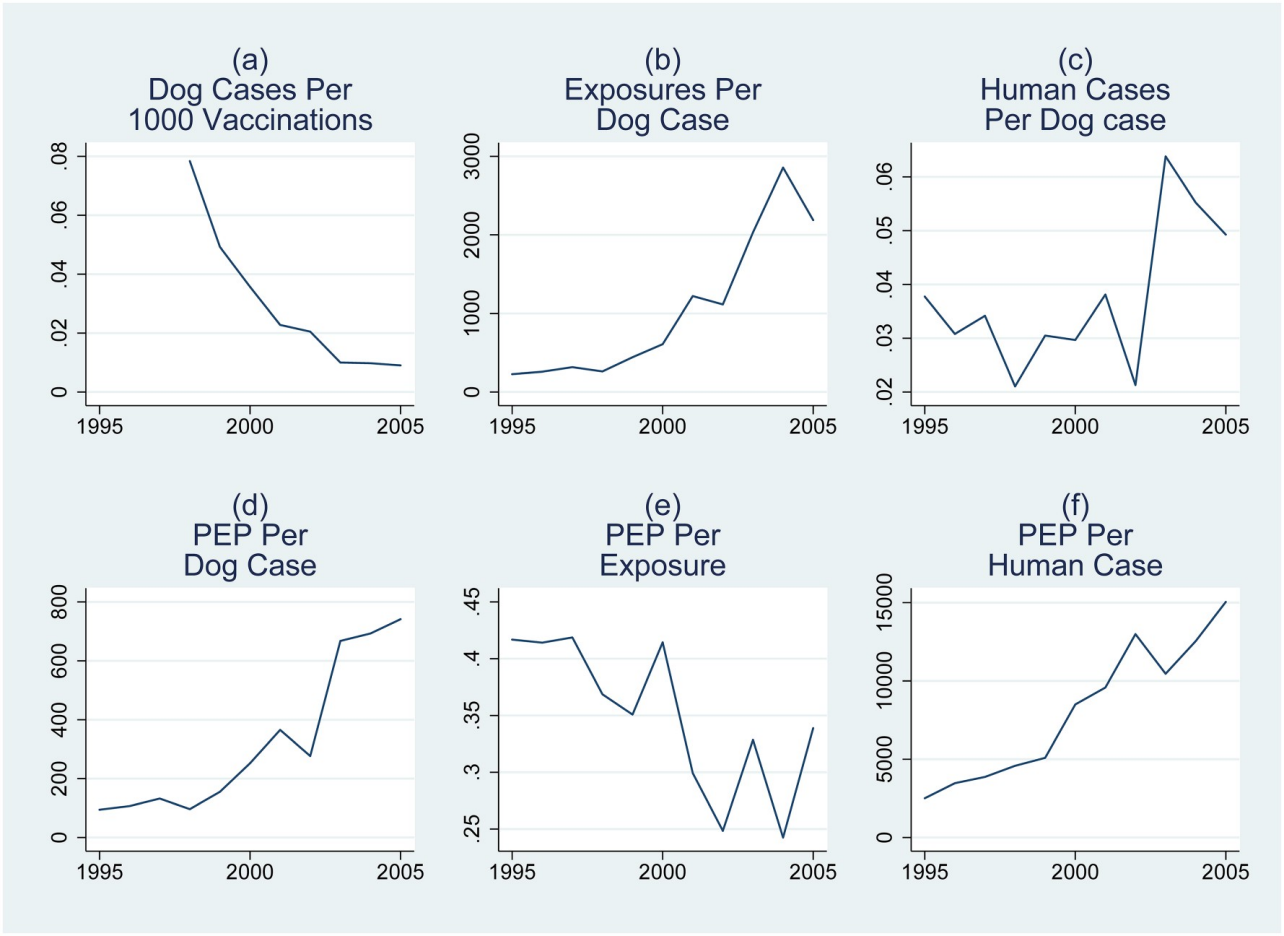
Time series of several ratios of focal variables aggregated over seven countries.

Fig 2 shows time trends for the five focal variables, by country and in aggregate. Fig 3 provides six panels, including dog rabies cases per dog vaccination, reported exposures per dog rabies case, human cases per dog case, PEP completions per dog case, PEP completions per exposure, and PEP completions per human rabies case.

Additional factors hypothesized to affect Reported Exposures and PEP Completions ($Z_{i,t-j}^{H}\;and\;Z_{i,t-j}^{P}$ in Fig 1) were collected from the World Bank Open Data website [23], including human Urban Population $\left(H_{i,t}^{u}\right)$ and Rural Population $\left(H_{i,t}^{r}\right)$, [per capita] Income (*I*_*i*,*t*_), [per capita] Health Expenditures (*X*_*i*,*t*_), and Out of Pocket [health expenditures] (*O*_*i*,*t*_). Additional data issues, including missing dog population data, two potential anomalies in the Brazilian data, and that reported exposures are not limited to dog bites are discussed in S1 File. Freire de Carvalho et al. (2018) [16], whose data overlap with ours to some degree, discuss some additional limitations of the data, mostly having to do with potential shortcomings of the reporting process for PEP. The limited sample size is among most pressing limitations of our study, but the overall trends in mass dog vaccination, dog and human rabies cases, and their relationship to reported exposures and PEP completions tend to be relatively robust.

### Empirical methods

We estimate five regressions: one each for Dog Vaccinations (*V*_*t*_), Dog Rabies Cases *$\left(R_{i,t}^{D}\right)$*, Reported Exposures (*E*_*i*,*t*_), PEP Completions (*P*_*i*,*t*_), and Human Rabies Cases $\left(R_{t}^{H}\right)$. The data are in the form of a panel of country-specific time series, so we use panel regression models for each. We hypothesize that Dog Vaccinations, Dog Rabies Cases, and Reported Exposures (*V*_*i*,*t*_, $R_{i,t}^{D}$, *E*_*i*,*t*_) exhibit some dynamic autoregressive relationship. We therefore use an Arellano-Bond panel estimator for these regressions for statistical consistency [24,25].

Reported Exposures and PEP Completions accumulate based on decisions driven by subjective rabies risk assessment and other underlying supply and demand factors. Further, Reported Exposures may be an endogenous determinant of PEP Completions, and PEP Completions may be an endogenous determinant of Human Rabies Cases. We therefore test for endogeneity of these variables for use as regressors in other regressions using a Durbin-Wu-Hausman Test. We fail to reject exogeneity of Reported exposures (*E*_*i*,*t*_) in the PEP Completions regression, and reject exogeneity of PEP Completions (*P*_*i*,*t*_) in the Human Rabies Cases regression. We therefore treat Reported Exposures as exogenous and use the original values for *E*_*i*,*t*_ in the PEP Completions regression, but use predicted values from the PEP Completions regression as an instrument in the Human Rabies Cases regression.

All dependent variables are non-negative count data and wide domains allow most to be treated as continuous variables. Exploratory analysis suggested that regression error distributions approximate a lognormal distribution for *V*_*i*,*t*_, $R_{i,t}^{D}$, *E*_*i*,*t*_, and *P*_*i*,*t*_, so we transformed each of these dependent variables into natural logarithms for estimation. Human rabies cases $R_{i,t}^{H}$ are smaller in number for each country/year than the rest. Specification search and tests lead us to use a Negative Binomial regression in this case. Consequently, parameter estimates of all continuous variables in each regression (including the $R_{i,t}^{H}$ regression) can be interpreted as elasticities (i.e. the percent changes in the dependent variable in response to a one percent change in the associated explanatory variable). Robust standard errors are applied throughout. Analysis was performed using Stata version 14.2.

Our theoretical model is built on concepts of causality (e.g. dog vaccination is a treatment that systematically leads to fewer dog rabies cases, *ceteris paribus*; a higher rate of PEP use per dog bite reduces the proportion of human deaths among dog bite victims). Further, the endogeneity of decision variables such as self-reporting and PEP uptake are accounted for in the empirical estimation in part to try to identify causality where our theoretical model suggests it. However, limitations of the sample frame and data (omitted variables, small sample, data collection errors) imperfect instruments for testing and addressing endogeneity, and any remaining specification errors suggest caution in inferring strict quantitative causality among the estimated relationships. While we use language of causation in the presentation of some of the results for expediency and clarity, especially in terms of treatment effects, implications of causality in our results should be inferred with caution. Additional methodological details and data limitations are provided in S1 File.

## Results and discussion

During 1995 through 2005, there was an increase in Dog Vaccinations in the seven countries, accompanied by a marked decrease in both Dog and Human Rabies Cases (Fig 2, thick top line; natural logarithmic scale), while Reported exposures and PEP completions per dog case, and per human case increased substantially (Fig 3b, 3d and 3f, respectively). These general patterns provide context for interpreting our regression results.

Results are presented in Table 3 for four regressions—Regression 1: Dog Rabies Cases $\left(R_{i,t}^{D}\right)$; Regression 2: Reported Exposures (*E*_*i*,*t*_); Regression 3: PEP Completions (*P*_*i*,*t*_); and Regression 4: Human Rabies Cases $\left(R_{t}^{H}\right)$. The discussion of results below is organized around the relationships among critical policy-relevant variables. Additional discussion of all individual regression results, including Dog Vaccinations, is provided in S1 File.

### Impact of dog rabies vaccination on dog rabies cases

Factors that drive dog rabies cases include current rabies prevalence in the host population, vaccination coverage, and transmission rates. Dog Rabies Cases regression results (Table 3, Regression 1) suggest that the number of dog rabies cases is positively related to the number of rabies cases in prior years, and negatively affected by prior vaccination activity. The results show that a 10% increase (decrease) in a year’s Dog Rabies Cases is associated with 5.6% more (fewer) cases in the following year (indicated by the coefficient of 0.56 associated with $\text{ln}\;\left(R_{t-1}^{D}\right)$).

The estimated effects of current year and last year’s vaccinations (ln(*V*_*t*_) and ln(*V*_*t*−1_)) are negative, but statistically weak. Vaccinations conducted two years prior (ln(*V*_*t*−2_)) has a significant but slightly smaller direct effect, with 10% more vaccinations resulting in a 3.1% decrease in dog rabies cases (corresponding to a parameter estimate of -0.31 associated with ln(*V*_*t*−2_)). As expected, current and prior year Dog Vaccinations negatively affect current Dog Rabies Cases. A χ^2^ test suggests that this set of vaccination variables are jointly significant at the 10% level (χ^2^(3) = 6.64, p = 0.084). The cumulative effect of vaccination over a three-year period is -2.30: a 10% increase in vaccinations decreases dog rabies over three years by 23% (see Equation (S8) in S1 File for details).

### Effects of dog rabies vaccination campaigns on reported human exposures

In contrast to declines in Dog Rabies Cases and Human Rabies Cases, there was an increase in Reported Exposures (Fig 2) in our sample period, resulting in a marked increase in reported human exposures per dog rabies case (Fig 3 panel (b)). Table 3, Regression 2 provides estimates of the effect on Reported Exposures of past exposure reports, current and recent rabies cases, a time trend, and dog and human populations.

Correlation across years in Reported Exposures could be expected because of stable reporting infrastructure including clinics/hospitals, health care providers, and available rabies-related information. There is a statistically strong correlation between current Reported Exposures and past Dog Rabies Cases: 10% fewer Dog Rabies Cases in the current year or prior year is associated with 1% fewer current Reported Exposures, suggesting that if all other factors were held constant, Reported Exposures would have declined along with declines in Dog Rabies Cases.

When the effects of current Dog Rabies Cases are controlled for in our regression framework, past Dog Rabies Cases should not change current exposures, and neither should last year’s Human Rabies Cases (as humans are terminal hosts). However, even while statistically controlling for dog rabies cases in Regression 2, the results show a positive relationship between current Reported Exposures and both *Dog* and Human Rabies Cases in the previous year. This suggests that past human and dog rabies cases are salient events that inform people’s subjective rabies risk assessment, and they act on the information by reporting current exposure events more often when there have been recent publicized dog and human rabies cases.

A time trend (measured in years from 1994) in logarithmic form (ln(*t*)) is included in Regression 2 to control for unobserved factors that may be driving exposure reporting. Its associated parameter estimate is strongly positive. Reported Exposures result from a complex mix of private decisions to seek care, including individuals’ assessment of risk given both case-specific (e.g. wound severity, behavior of and familiarity with the dog, knowledge of dog vaccination status) and non-case-specific factors (e.g. formal and informal information about rabies circulating in the community). We hypothesize that the increase in Reported Exposures is at least partly due to increasing awareness of rabies during this period of active dog vaccination campaigns [26].

### Effects of dog rabies vaccination and other factors on PEP completions

Although the actual risk of human rabies decreased during the period due to the declines in dog rabies, there was no clear reduction in PEP Completions. Consequently, given the marked reduction in both Dog and Human Rabies Cases during the period, there was a substantial increase in both the number of PEP Completions per Dog Rabies Case and per Human rabies Case (Fig 3d and 3f, respectively).

PEP Completions (Table 3, Regression 3) is a reduced-form regression representing both demand for and supply of PEP (further discussion in S1 File). As with exposure reporting, investing in PEP entails a series of complex and often subjective risk and cost-benefit assessments. Contemporaneous Reported Exposures had a positive but weak relationship to PEP Completions. The mean number of Reported Exposures was two and a half times higher than that of PEP Completions (Table 2), suggesting that a clinical decision to forgo PEP occurred for many of the reported exposures. There is not a significant relationship between current PEP Completions and current Dog Rabies Cases, but there is a positive relationship between current PEP Completions and prior years’ Dog Rabies Cases: a 1.3% increase in PEP Completions per 10% increase in lagged Dog Rabies Cases. Similarly, PEP Completions is positively related to lagged human rabies cases, with an estimated 3% increase in a current year’s PEP use in response to 10% more human rabies cases in the previous year. In comparison with the Reported Exposures regression results, we hypothesize that increased PEP demand is responding to changes in subjective risk assessments which are informed by prior dog and human rabies outbreaks.

Per capita Income (*I*_*i*,*t*_) is strongly associated with PEP Completions (Table 3, Regression 3). A 1% percent increase in Income is associated with a 2% increase in PEP Completions, conditional on other factors. PEP Completions is also positively associated with public per capita Health Expenditures (*X*_*i*,*t*_), and negatively associated with the proportion of healthcare borne Out of Pocket (*O*_*i*,*t*_). The latter is consistent with the income effect in relation to PEP costs, and both results are consistent with access and availability to PEP being perceived to be within the remit of the public health care systems in Latin America.

### Effect of dog rabies vaccinations and PEP use on human rabies deaths

The incidence of human rabies cases (deaths) is determined jointly by exposure to a rabid animal and PEP completion, mediated through exposure reporting. PEP use is an endogenous factor affecting human rabies cases because PEP will be completed when risk is high, which in itself would lead to a positive correlation between PEP use and human rabies cases in aggregate data even though PEP use reduces the incidence of death in the exposed population. Using the two-stage instrumental variable method described, a 10% increase in PEP Completions decreases Human Rabies Cases (death) by 7%, all else constant (Table 3, Regression 4). Without implementing the instrumental variable approach, the estimated impact is spuriously positive (0.42, p = 0.016), reflecting the use of PEP when risk would be highest. Conditional on PEP Completions, Human Rabies Cases are positively related to Dog Rabies Cases, with elasticity of 0.63. Thus, a 10% increase in Dog Rabies Cases is associated with a 6.3% increase in Human Rabies Cases, holding PEP Completions constant.

The effect of Dog Vaccinations on Human Rabies Cases can be estimated through its effects on Dog Rabies Cases while accounting for changes in PEP Completions as

$$\begin{array}{ll} \frac{\%\Delta R^{H}}{\%\Delta V} & =\left(\frac{\partial \;\text{ln}\left(R^{H}\right)}{\partial \;\text{ln}\left(R^{D}\right)}+\frac{\partial \;\text{ln}\left(R^{H}\right)}{\partial \;\text{ln}\left(P\right)}\frac{\partial \;\text{ln}\left(P\right)}{\partial \;\text{ln}\left(R^{D}\right)}\right)\times \frac{\partial \;\text{ln}\left(R^{D}\right)}{\partial \;\text{ln}\left(V\right)}\\ & =\left(0.63+\left(-0.71\times \left(0.13-0.01\right)\right)\right)\times \left(-2.30\right)=-1.25. \end{array}$$ (1)

The numerical values are based on regression parameter estimates (Table 3, Regressions 3 and 4), where 0.12 = (0.13 − 0.01) is the total impact over two years of Dog Rabies Cases (*R*^*D*^) on PEP Completions, and -2.3 is the three-period (long-run) effect of Dog Vaccinations on Dog Rabies Cases. Thus, a 10% increase in Dog Vaccinations reduces human deaths by 12.5% over the subsequent three years.

The marginal effect of Dog Vaccinations on Human Rabies Cases is $\frac{\partial R^{H}}{\partial V}\;=\;\frac{\%\Delta R^{H}}{\%\Delta V}\frac{V}{R^{D}}\;=\;\frac{\partial \text{ln}\;R^{H}}{\partial \text{ln}\;V}\frac{V}{R^{D}}$. Consider the conditions at the beginning and end of our sample: In 1995, about 100 Human Rabies Cases were reported in these seven countries. By 2005, this number was under 20. While we do not have vaccination data for 1995–1997, the subsequent years’ data indicate that an average of 3.5 million dogs were vaccinated annually in these countries combined; an average of 33 million per year prior to 2000 and about 37 million from 2000 to 2005. Consequently, the marginal effectiveness of vaccination for reducing human deaths for early and late in the sample were:

$$\begin{array}{l} \frac{\partial R^{H}}{\partial V}{|}_{early}=\frac{\partial \;\text{ln}R^{H}}{\partial \;\text{ln}V}\times \frac{V\left(early\right)}{R^{H}\left(early\right)}=-1.25\times \frac{100}{33}=\;3.8\\ \Rightarrow \;3.8\;\text{human}\;\text{lives}\;\text{saved}\;\text{per}\;\text{million}\;\text{vaccinations}\;\text{per}\;\text{year}, \end{array}$$ (2)

$$\begin{array}{l} \frac{\partial R^{H}}{\partial V}{|}_{late}=\frac{\partial \;\text{ln}R^{H}}{\partial \;\text{ln}V}\times \frac{V\left(late\right)}{R^{H}\left(late\right)}=-1.25\times \frac{20}{37}=\;0.68\\ \Rightarrow \;0.68\;\text{human}\;\text{lives}\;\text{saved}\;\text{per}\;\text{million}\;\text{vaccinations}\;\text{per}\;\text{year}. \end{array}$$ (3)

The difference between effectiveness early and late in the sample statistically illustrates the “tribulations of the last mile” described in Del Rio Vilas et al. [14].

The percentage change in PEP Completions in response to a percentage change in Dog Vaccinations is embedded in Eq (1) as:

$$\begin{array}{ll} \frac{\text{\%}\partial P}{\text{\%}\partial V} & =\frac{\partial \;\text{ln}\left(P\right)}{\partial \;\text{ln}\left(R^{D}\right)}\times \frac{\partial \;\text{ln}\left(R^{D}\right)}{\partial \;\text{ln}\left(V\right)}\\ & =0.12\times -2.3=-0.276 \end{array}$$ (4)

This implies that a 10% increase in dog vaccinations leads to a 2.8% decrease in PEP use. For Mexico, the average annual number of dog vaccinations in the period was approximately 14 million and PEP Completions averaged approximately 17,000 per year. One thousand dog vaccinations reduces PEP completions by 0.34 units, or equivalently, one PEP completion is avoided per approximately 3,000 dog vaccinations, all else constant. This amounts to a net effect of dog vaccination, which decreases the risk of rabies given an exposure, and we speculate also increases public awareness of rabies as a threat to health and of PEP as an option for alleviating the rabies risk following a potential exposure.

Income affects the number of human deaths through its effect on PEP use. The percent change in Human Rabies Cases with a percent change in Income is

$$\begin{array}{ll} \frac{{\%\Delta \text{R}}^{\text{H}}}{\%\Delta \text{I}} & =\frac{\partial \;\text{ln}\left(R^{H}\right)}{\partial \;\text{ln}\left(P\right)}\times \frac{\partial \;\text{ln}\left(P\right)}{\partial \;\text{ln}\left(I\right)}\\ & =-0.71\times 2.05=-1.46, \end{array}$$ (5)

suggesting that a 1% increase in Income is associated with 1.46% fewer human deaths (14.6% for a 10% increase in income), an effect slightly larger in magnitude than the estimated effect of dog vaccination on human rabies cases (-1.25%, or -12.5% for a 10% increase in vaccination). Per capita annual income (2011 base) ranges from approximately US$3,000 (Nicaragua) to US$15,000 (Mexico), and the mean number of deaths ranges from 0.3 (Nicaragua) to 19.5 (Brazil), respectively. Taking Brazil as an example, with a mean per capita income of about $11,000 and an average of 19.5 deaths per year in the sample, an increase in per capita income of about $1,000 per year would reduce human deaths by about 2.6 people per year $\left(-1.46\times (\frac{19.5}{11})=2.6\right)$. Given an average number of PEP Completions for Brazil of 22,300/year, an increase of per capita income of $1,000 would also be associated with about 40 more PEP completions, all else constant.

## Conclusion

Elimination of human death due to rabies by 2030 requires reduction of the primary transmission risk (rabid dogs) through vaccination, prompt exposure reporting, and universal PEP access. The Latin American example illustrates how coordinated multi-national investment can substantially reduce dog and human rabies over time [27]. However, there has been justifiable concern over an apparent positive connection between successful dog vaccination campaigns and increased expenditures on PEP, because if this connection holds the costs of rabies elimination that result from increased PEP use may be high. PEP demand is driven in part by awareness, salience, and perceptions of rabies risk. Our results suggest that as dog and human rabies cases and risk decline, reported exposures and PEP use tends to decline as well, all else constant. The overall increases in exposures and PEP use appear instead to be driven by other factors, such as awareness and salience of rabies as a risk, which are likely to increase with rabies control efforts and real income and public health investments.

If lessons from Latin America hold, potential decrease in dog and human rabies in Africa and Asia through national and regional MDV may not necessarily generate a proportional reduction in PEP demand. These findings about exposure reporting and PEP demand underscore the importance of promoting and supporting integrated bite case management approaches [28,29,30], such as clinical algorithms, which allow systematic clinical assessment of potential exposures PEP and treatment determinations following suspect dog bite exposures. Effective, continuously improving algorithms, based on confirmed dog vaccination and health history, quarantine, and laboratory testing, have the potential to reduce costly false-positive PEP prescriptions and false negatives leading to human death. The lessons from Latin America highlighted in our study emphasize the need for ongoing evaluation of human exposure data and PEP use relative to reduction in canine rabies due to MDV and understanding of the drivers of risk perception in addition to actual risk. These factors will be critical in sustaining the national investments, predominantly by low- and middle-income countries, required to meet the 2030 goal of no human deaths due to rabies.

## Supporting information

S1 File Supplementary materials. S1 File contains supplementary information in the following sections: S1. Theory; S2. Data; S3. Empirical Approach; and S4. Results; S5. References for Supplementary Materials.
(DOCX)

S2 File Spanish language abstract. S2 File contains the submitted abstract translated into the Spanish language.
(DOCX)

S3 File Data. S3 File is a Microsoft Excel file that contains all and only the data used in the analysis. The first row of the data file provides the variable abbreviations consistent with their use in the article (except without the use of superscripts).
(XLS)
