# Supplementary material for: Healthcare demand in response to rabies elimination campaigns in Latin America
Source: PLoS Negl Trop Dis. 2019 Sep 26;13(9):e0007630. doi: 10.1371/journal.pntd.0007630 (PMC6762069; doi:10.1371/journal.pntd.0007630)
Supplement: S1 File — contains supplementary information in the following sections: S1. Theory; S2. Data; S3. Empirical Approach; and S4. Results; S5. References for Supplementary Materials. (DOCX) [file pntd.0007630.s001.docx]

# Supplementary Materials

Title: Healthcare demand in response to rabies elimination campaigns in Latin America

Short title: Rabies risk and healthcare demand

Jonathan Yoder^*,1,2^, Elisabeth Younce^2^, Felix Lankester^2^, Guy H. Palmer^2^

^*^Corresponding Author

[yoder@wsu.edu](mailto:yoder@wsu.edu)

<https://orcid.org/0000-0002-9049-9591>

^1^School of Economic Sciences

PO Box 6210

203B Hulbert Hall

Washington State University

Pullman, WA 99164-6210

^2^Paul G. Allen School for Global Animal Health

PO Box 647090

240 SE Ott Road

Washington State University

Pullman, WA 99164-7090

# S1. Theory

Here we develop a mathematical representation of relationships illustrated in Fig. 1 in the main text as a system of equations that supports the regression analysis. The five equations of interest relate to Dog Vaccinations$\left( V_{i,t} \right)$*,* Dog Rabies Cases $\left( R_{i,t}^{D} \right)$, Reported [human] Exposures $\left( E_{i,t} \right)$, PEP Completions $\left( P_{i,t} \right)$, and Human Rabies Cases $\left( R_{t}^{H} \right)$, and can be written in general terms as:

|  | $V{}_{i,t}=f\left( \boldsymbol{V}_{i,L\left( t \right)}\boldsymbol{,}\boldsymbol{R}_{i,L\left( t \right)}^{D}\boldsymbol{,}\boldsymbol{Z}_{i,t}^{V} \right)$ | (S1) |
| --- | --- | --- |
|  | $R_{i,t}^{D}=f\left( \boldsymbol{R}_{i,L\left( t \right)}^{D},\boldsymbol{V}_{i,L\left( t \right)},\boldsymbol{Z}_{i,t}^{D} \right)$ | (S2) |
|  | $E_{i,t}=f\left( R_{i,t}^{D}, \boldsymbol{Z}_{i,t}^{E} \right)$ | (S3) |
|  | $P_{i,t}=f\left( E_{i,t},\boldsymbol{Z}_{i,t}^{P} \right)$ | (S4) |
|  | $R_{i,t}^{H}=f\left( E_{i,t},P_{i,t},\boldsymbol{Z}_{i,t}^{H} \right)$ | (S5) |

Where:

- $i$and $t$ are country and time indexes; *L(t)* represents a general lag structure for the associated variable, and bold text represents an algebraic vector of variables.
- $V_{i,t}$ is the number of Dog Vaccinations against rabies in country *i* and year *t*. $\boldsymbol{V}_{i,L\left( t \right)}$ represents one or more lagged values of $V_{i,t}$
- $R_{i,t}^{D}$ is the number of Dog Rabies Cases. $\boldsymbol{R}_{i,L\left( t \right)}^{D}$ represents lagged values of $R_{i,.}^{D}$
- $E_{i,t}$ is the number of Reported [human] Exposures
- $P_{i,t}$ is the number of PEP Completions
- $R_{i,t}^{H}$ is the number of Human Rabies Cases (deaths)
- $\boldsymbol{Z}_{i,t}^{V}$ are exogenous supply and demand factors affecting $V_{i,t}$
- $\boldsymbol{Z}_{i,t}^{D}$ are exogenous factors affecting $R_{i,t}^{D}$
- $\boldsymbol{Z}_{i,t}^{E}$ are exogenous factors affecting $E_{i,t}$
- $\boldsymbol{Z}_{i,t}^{P}$ are exogenous supply and demand factors affecting $P_{i,t}$
- $\boldsymbol{Z}_{i,t}^{H}$ are exogenous factors affecting $R_{i,t}^{H}$

Below we provide some basic supplementary background theory to conceptually support our empirical specifications.

**Dog Vaccinations** $\left( \boldsymbol{V}_{\boldsymbol{i,t}} \right)$. The number of dogs vaccinated in a country and year (Equation S1) represents the coincidence of public administrative supply decisions, and public demand and participation in mass dog rabies campaigns. We include lagged values of Dog Vaccinations to capture both a country’s historic propensity for vaccination and their decisions about recent vaccination investments. Lagged values of Dog Rabies Cases are included as an indicator of rabies outbreak risk, and lagged values of Human Rabies Cases, which are hypothesized to be correlated with dog vaccination demand through a policy process. We include Dog Population to represent the scale of the target dog population. Actual public expenditures or availability of dog rabies vaccination stocks on hand are not available. We therefore include per capita Income and per-capita public Health Expenditures as proxies for supply-side factors affecting a country’s general propensity to invest in rabies vaccination activities. We relegate the vaccination regression and discussion of it to Supplementary Materials (section S4), because although it is related to our analysis, it is not central to the focus of our primary results, and our data for empirically estimating a regression explaining vaccination activity is especially limited in both the time series length and the availability of supply-side drivers.

**Dog Rabies Cases *(***$\boldsymbol{R}_{\boldsymbol{t}}^{\boldsymbol{D}}$***)*.** The number of Dog Rabies Cases (Equation S2) is hypothesized to be a function of recent Dog Rabies Cases, recent Dog Vaccinations activity, Dog Population (which together define the vaccination coverage rate), and conditions that affect transmission rates. These determinants are generally consistent with hypothesized drivers of canine rabies cases in the epidemiological models of rabies [1-3].

**Human exposures *(***$\boldsymbol{E}_{\boldsymbol{i,t}}$***).*** The number of human exposures to suspect dogs (Equation 9) is defined as the number reported cases for which there is some risk of rabies based on initial assessment by a clinician, given that a bite victim visits a clinic. Based on a random utility model [4,5], a victim of suspect dog contact (or her agent) chooses to visit a clinic after a dog bite if her expected utility of doing so is larger than not. Let $\pi_{i,t}^{E}\left( \mathbf{Z}_{i,t}^{E_{\boldsymbol{r}}} \right)\in\left[ 0,1 \right]$ be the reporting rate, which is affected by factors $\mathbf{Z}_{i,t}^{E_{\boldsymbol{r}}}$. Define $E^{c}\left( \boldsymbol{Z}_{i,t}^{E_{c}} \right)$ as the number of human *c*ontacts (identified by script $c$) with dogs at time $t$ in country *i* that might lead to rabies infection, whether reported or not, including cases where rabies status of the dogs is uncertain. $\mathbf{Z}_{i,t}^{E_{c}}$are factors that affect this number, such as dog and human populations and metrics that describe how they interact. Then let

|  | $E\left( \mathbf{Z}_{i,t}^{E} \right)=\pi_{i,t}^{E}\left( \mathbf{Z}_{i,t}^{E_{r}} \right)\times E^{c}\left( \mathbf{Z}_{i,t}^{E_{c}} \right)$ | (S6) |
| --- | --- | --- |

be the number of reported exposures in country *i* at time $t$, where $\boldsymbol{Z}_{i,t}^{E}=\left[ \begin{matrix} \boldsymbol{Z}_{i,t}^{E_{r}} & \boldsymbol{Z}_{t}^{E_{c}} \end{matrix} \right]$. Equation (S6) can be interpreted as representing the aggregate demand for clinic visits in response to suspect dog contact, and is a function both of the factors that affect individuals’ decisions as well as the scale of human and dog populations and the nature of their interactions. In our exposure regression model we use past exposures, past dog rabies cases, urban and rural human population, and dog population as proxies for these factors.

**PEP Completions**$\left( P_{i,t} \right)$. Several articles develop PEP decision-tree analyses that provide additional detail on PEP decisions [e.g. 6-8]. PEP demand at the individual level through the lens of a Random Utility Model depends on a risk assessment by the victim or their agents, who may include parents, health care workers, or others. Decisions to administer PEP depends in part on the nature of the exposure to a potentially rabid dog, their understanding of rabies PEP use guidelines, their perceived liability associated with not prescribing PEP in a suspect case, etc. PEP is expensive, and the cost (both in terms of time and money) can be a deterrent to investment in PEP. Aggregating up from individual PEP decisions, the number of PEP completions can be defined in terms of the number of reported exposures, and the rate of PEP completion per reported exposure:

|  | $P\left( \mathbf{Z}_{i,t}^{P} \right)=\pi_{i,t}^{P}\left( \mathbf{Z}_{i,t}^{P_{\pi}} \right)\times E\left( \mathbf{Z}_{i,t}^{E} \right)$, | (S7) |
| --- | --- | --- |

Where $\pi_{i,t}^{P}\left( \mathbf{Z}_{i,t}^{P} \right)$ is the rate of PEP Completion per Reported Exposure, $E\left( \mathbf{Z}_{i,t}^{E} \right)$, from Equation [(S6)](#_bookmark10), and $\mathbf{Z}_{i,t}^{P}=\left[ \mathbf{Z}_{i,t}^{P_{\pi}}\mathbf{Z}_{i,t}^{E} \right]$. $P\left( \boldsymbol{Z}_{i,t}^{P} \right)$ represents aggregate PEP demand. PEP treatment is expensive, and so PEP demand is likely to depend on income and out-of-pocket expenses of treatment. Our PEP regression uses past dog and human cases as proxies for rabies risk conditional on reported exposures. It also includes human urban and rural population numbers, income, public health expenditures for each country and year, and out-of-pocket health expenditures (see Tables 1-3).

**Human Rabies Cases** $\left( R_{i,t}^{H} \right)$. Human cases (Equation S5) imply human deaths and occur when an exposed individual developed rabies after an infection and PEP was not administered on time for any reason. A failure to administer PEP can follow from a failure of the victim to identify an exposure, a failure or sufficient delay in pursuing treatment after an exposure, or a decision to forego PEP in agreement with or despite clinical advice. Our Human Rabies Cases regression includes PEP usage (accounting for endogeneity as described in the Empirical Methods section in the main text and S3. Empirical Approach, below), past rabies cases to proxy for perceived risk, and reported exposures and human urban and rural populations to account for scale.

# S2. Data

Data, sources, descriptions, and summary statistics and illustrative figures are provided in the main text. Here we provide a brief description of two potential anomalies in the data and how we addressed them. Fig. [2](#_bookmark16) shows individual time series for the seven countries for four variables, showing that Brazil dominates the other countries in terms of rabies activity. This is not necessarily surprising given its relative size.

One potential anomaly is the large increase in the number of dog rabies cases in Brazil between 1995 and 1998. These are the only data we know of to represent dog rabies cases, and because they come at the beginning of the time series and data are missing for vaccinations until 1998, we have no basis for assessing the veracity of this large increase during this period.

Fig. 2 (panel 3) shows an abrupt drop in Reported Exposures in 2002 and 2003, followed by a rebound. It is clear from the figure that this abrupt drop in the aggregate time series is attributable entirely to Brazil. It might not be surprising to see large fluctuations in factors being driven in part by public health care stocks of vaccinations and PEP, or relatively large fluctuations in variables with low sample numbers. Further, there is also an apparent contemporary drop in Human Rabies Cases in Brazil. However, this drop in Reported Exposures for Brazil is more suspect because for this variable in particular, the source of this precipitous drop and recovery seems much more likely to be a reporting anomaly rather than a representation of actual swings in exposures or reporting rates. As a result, in preliminary regressions, we replaced the anomalous values for 2002 and 2003 with linearly interpolated values and used these in all regressions (shown in Fig. 2, middle panel near the top, as a dotted line connecting Brazilian Reported Exposures between 2001 and 2004). The results of all regression were qualitatively and quantitatively similar, presumably in part because these represented only two data points among 60, and because while large in absolute value, the fluctuation is not as extreme in percentage terms. Because the differences were minor, we use the original data in the reported analysis.

# S3. Empirical Approach

The data characteristics inform the econometric methods used to estimate the relationships embodied in Equations S[1](#_bookmark3) – S[5](#_bookmark6), including the panel structure of the data along with the dynamic (autoregressive) nature of several of the relationships, the empirical distribution of regression residuals, and potential endogeneity of Reported human exposures in PEP demand and PEP in human rabies cases. We discuss these issues in turn.

Because the data are in the form of a panel of seven country-specific time series, we utilize panel regression models for all regressions but the Human Rabies Cases regression, based on model specification tests. All dependent variables are non-negative count data. The domain of most of them are wide enough that we treat them as continuous variables. Based on exploratory analysis that suggests that regression errors tend to approximate a lognormal distribution, we transform each variable into natural logarithms for estimation. One consequence of this specification is that regression parameter estimates can be interpreted as elasticities: percent changes in the dependent variable resulting from a percentage change in the associated independent variable. Human Rabies Cases $R_{i,t}^{H}$ is unique among these regressands in that it takes smaller values than the other dependent variables, and 27% of observations take the value of zero, the consequences of which we discuss below.

Vaccination, dog rabies, and human exposure ($V_{i,t}$, $R_{i,t}^{D}$,$E_{i,t}$) are all hypothesized to exhibit some dynamic autoregressive relationship. We therefore used an Arellano-Bond panel estimator [9,10] for these regressions. Autoregression in first-differenced errors was identified in all three of these regressions, so we used a lagged first difference of the dependent variable [11], and a double-lag difference was used as an instrument, as necessary for consistency. The null hypothesis Sargan’s test for valid over-identifying restrictions [12,13] was not rejected in all three of these specifications (minimum p > 0.49). Robust (sandwich) standard errors are used as parameter covariance estimator.

The PEP regression represents a reduced form model of drivers of PEP use, driven by underlying PEP supply and demand. A Random Effects model was strongly rejected based on a Durbin-Wu-Hausman Test (χ^2^ (6) = 39, p-value < 0.0001). A joint test that country-specific fixed effects are not different was rejected (F(6,52) = 12.27, p-value < 0.0001), so a pooled model was rejected. The reported estimates are therefore based on a fixed effects model. Reported exposures were included in the PEP regression, but may be jointly determined and endogenous with respect to PEP, a characteristic that must be addressed for unbiased and consistent parameter estimation. We tested for endogeneity of reported exposures in the PEP regression and found that a Durbin-Wu-Hausman test failed to reject exogeneity of ln(*E*) (p-value *=* 0.91). We therefore used the original values of $E_{i,t}$ as a regressor in the regression PEP regression.

The Human Rabies Cases $\left( R_{i,t}^{H} \right)$ regression has two additional complications. First, there were fewer human rabies cases per country/year, and 27% of observations take the value zero. Second, PEP is likely to be an endogenous regressor in the human rabies regression because PEP acquisition is driven by many of the same risk factors that drive human rabies cases.

To address the small integers and a large number of zeros, several models were considered, including (a) Fixed and Random Effects Poisson and Zero-Inflated Poisson (b) Fixed and Random Effects Negative Binomial and Zero-Inflated Negative Binomial models, and (c) Fixed and Random Effects Tobit models.

Durbin-Wu-Hausman tests for Fixed versus Random Effects, and for endogeneity of $P_{i,t}$, were performed. Tests for over-dispersion to compare Poisson and Negative Binomial regressions, as well as AIC and BIC levels to compare zero-inflated Poisson and Negative Binomial models were also performed. Based on these comparisons, Poisson, Zero-Inflated Poisson, and Zero-Inflated Negative Binomial regressions were rejected based on Akaike and Bayesian Information Criteria (the Vuong Test until recently has been widely used to test for zero-inflated models over their non-zero-inflated counterparts. This recently has been found inappropriate [14]). Various specifications of Tobit models (Fixed, Random Effects, and Pooled) were considered and found to be dominated by Negative Binomial models based on AIC and BIC criteria. Random effects Negative Binomial specifications were rejected based on a Durbin-Wu-Hausman test (p-value *=* 0.088).

A Fixed Effects Negative Binomial provided similar results to the Random Effects model, but many of the fixed effects were not statistically different from zero, which inflated the variance estimates. Therefore, a compromise strategy was applied that used a pooled Negative Binomial (neither Fixed nor Random Effects), with a dummy variable (BMP*)* to represent Brazil, Mexico, and Peru, which were significantly different in the number of human cases and suggested by a sequential fixed effect deletion process. While this compromise improved precision, the parameter estimates themselves were quite robust to various fixed-effects specifications. Random and Fixed Effects Tobit regressions also provided similar elasticities. The final reported regressions for Human Rabies Cases $\left( R_{i,t}^{H} \right)$ is a pooled Negative Binomial regression that includes a targeted indicator variable (*BMP)* to account for fixed effects associated with Brazil, Mexico, and Peru. Because this is a Negative Binomial Regression and all continuous regressors are log-transformed, the parameter estimates represent elasticities as in the other regressions.

A Durbin-Wu-Hausman test leads to rejection of exogeneity of $P_{i,t}$ in the Human Rabies Cases regression (p-value *=* 0.0055), so a two-stage Instrumental Variables approach was used in the final model. The instruments were those included in the PEP regression equation but excluded from the human rabies case equation. The predicted value from the PEP regression was included in place of the original variable, and a consistent estimate of the parameter covariance estimates was generated by bootstrap resampling [15]. All regressions are performed using Stata*Q*c Version 14.2.

# S4. Results

Results are presented in Table 3 in the main text for all regression except for the Dog Vaccinations regression. The discussion of results in the main text is not comprehensive, but is limited to specific topics of interest to maintain focus. We therefore provide some additional interpretation and discussion of regression results in this supplementary section, including the Dog Vaccination results, which may be of interest but were omitted from the main text to maintain focus on our objectives.

As noted above and in the main text, in all but the Human Rabies Cases regression the regressions are linear-in-logarithms, and the natural logarithm of all continuous variables are used for both the dependent and independent variables. The Human Rabies Cases regression is a Negative Binomial Maximum Likelihood regression, with the logarithms of the independent variables. The consequence is that all parameter estimates in all models (including $R_{i,t}^{H}$) can be interpreted as elasticities: $\frac{\partial\ln\left( y \right)}{\partial\ln\left( x \right)}=\frac{\partial y}{\partial x}\frac{x}{y}$, where *y* is the (original) dependent variable, and *x* is an (original) independent variable. The marginal effect (a unit change in *y* with respect to a unit change in *x*) is calculated by multiplying the elasticity by $\frac{y}{x}$, such that $\frac{\partial y}{\partial x}=\frac{\ln\left( y \right)}{\ln\left( x \right)}\frac{y}{x}=\beta\frac{y}{x}$, where $\beta$ is the regression parameter associated with $x$.

## Dog Vaccinations

Table [S1](#_bookmark21) reports the Dog Vaccinations regression. The results show that there tends to be a statistically strong negative relation between past vaccination and current vaccination. Holding dog populations constant, countries who have vaccinated more in recent years tend to vaccinate less in the current year, suggesting cyclicality in vaccination. None of the other coefficients are statistically significant on their own at conventional test sizes, and neither Dog Rabies Cases nor Human Rabies Cases are jointly significant (p-values for joint tests were p-value=0.71 and p-value=0.36, respectively). Although not statistically strong, the regressions suggest that if anything, more past human rabies cases and higher public health expenditures tend to be associated with higher vaccination activity. However, Dog Population $\left( D_{i,t} \right)$ and per capita Income $\left( I_{i,t} \right)$ are weakly negatively related to current vaccination activity.

| Table S1: Dog Vaccinations | | | | |
| --- | --- | --- | --- | --- |
| Dependent variable: $\ln\left( V_{i,t} \right)$; N=39, χ^2^ = 3192 | | | | |
| variable | coefficient | std. err. | t-stat. | p-value |
| $\ln\left( V_{t-1} \right)$ | -1.076*** | 0.224 | -4.79 | 0.000 |
| $\ln\left( V_{t-2} \right)$ | -0.741*** | 0.203 | -3.64 | 0.000 |
| $\ln\left( R_{t-1}^{D} \right)$ | 0.004 | 0.132 | 0.03 | 0.979 |
| $\ln\left( R_{t-2}^{D} \right)$ | -0.086 | 0.11 | -0.78 | 0.437 |
| $\ln\left( R_{t-1}^{H} \right)$ | 0.187 | 0.145 | 1.29 | 0.196 |
| $\ln\left( R_{t-2}^{H} \right)$ | 0.148 | 0.104 | 1.42 | 0.155 |
| $\ln\left( D \right)_{t}$ | -1.113 | 1.158 | -0.96 | 0.336 |
| $\ln\left( I_{t} \right)$ | -3.338 | 3.594 | -0.93 | 0.353 |
| $\ln\left( X_{t} \right)$ | 1.017 | 1.479 | 0.69 | 0.492 |
| $\mathrm{Constant}$ | 54.547 | 34.291 | 1.59 | 0.112 |
| ***p*<*0.01, **p*<*0.05, *p*<*0.1 | | | | |

## Dog rabies cases

As discussed in the main text, Table [3](#_bookmark23) shows that current Dog Rabies Cases is statistically positively related to the number of Dog Rabies Cases in the prior year: a 10 % increase (decrease) in a year’s cases is associated with 5.6% more (less) cases in the following year.

Current and prior year vaccinations are negatively and weakly correlated with current Dog Rabies Cases. The number of Dog Vaccinations delivered in the previous two years has a statistically strong negative impact on current rabies cases. A χ2 test suggests that this set of vaccination variables are jointly significant at the 10% level (χ^2^(3) =6.64, *p*=0.084). Ten percent more vaccinations in the prior year is associated with a nearly 6% reduction in rabies cases. Vaccinations two years prior has a slightly smaller direct effect (5.08% decrease for 10% more vaccinations). It can be shown [*16*] that the total effect of vaccination on rabies cases over its estimated three-year direct impact period is

| $\frac{-\left( 0.231+0.475+0.307 \right)}{\left( 1-0.560 \right)}=-2.30,$ | (S8) |
| --- | --- |

which means that taking account of indirect effects over two years, a 1% increase in vaccinations decreases dog rabies cases by an estimated 2.3%. This value is used in the main text in Equations 3 and 4 to estimate the relationship between dog vaccinations and PEP use, and human rabies cases.

One might expect the number of dogs to be positively related to the number of dog rabies cases, but the contrary seems to be true in this sample. That said, the number of dogs varies little within countries, and the within-country variation in the data is suspect, so the dog population estimates should probably be taken as mostly a cross-country effect. Further, as noted in the Data section, we imputed some missing values using a preliminary regression. There is also substantial concern over the quality of the dog population estimates (supporting about an hour’s worth of discussion at the REDIPRA 16 meetings in Guatemala City in November 2017).

## Reported human exposure to suspect dogs

Table 3 Regression 2 in the main text provides results of a regression of Reported Exposures as a function of past Reported Exposures, current and recent Dog Rabies Cases, a time trend, and dog and human populations numbers. There is a positive but small and weak correlation with last year’s Reported Exposures, and an unintuitive negative autocorrelation with a two-year lag, according to the results in Table [5](#_bookmark26). When the suspect drop in Brazilian Reported Exposures is replaced with interpolated values for the years 2002 and 2003, the past year’s effect becomes larger and more strongly positive (and statistically significant), but the long-run effect of the two lags is still strongly negative. Either way, both results suggests that there is some cyclicality in Reported Exposures, all else constant.

There is a statistically strong correlation between current Reported Exposures and past Dog Rabies Cases. A 10% higher number of rabies cases this year or last year is associated with about a 1% increase in reported exposures this year. Based on a sample mean of nearly reported 100,000 exposures per country per year and about 200 canine rabies cases in our sample, this means that 20 more rabies cases two years ago would lead to a total of about 100 new reported exposures over two years. We interpret the impacts of past rabies cases on current reported exposures as potentially due to their impact on awareness about and salience of rabies risk in the human population due to rabies outbreaks.

If rabies cases were the only type of information event driving reported exposures, Reported Exposures might go down over time. However, as shown in Fig. [3](#_bookmark17), the number of Reported Exposures per Dog Rabies Case has been increasing over time instead. We hypothesize that this is in part due to increasing clinical and public awareness of rabies during this period of active vaccination campaigns. We therefore include the natural logarithm of time (in years from 1994).

All else constant, one would expect to see more exposures given more dogs and more people. The coefficient on dogs (ln($D_{i,t}$)) is instead negative, though not statistically significant at conventional levels. Further, larger human populations — especially urban populations — are associated with fewer exposures, holding dog populations constant, which is also somewhat unexpected. However, because we cannot distinguish between rural and urban dog populations, it could be that these results may be picking up some negative correlation between urban population densities and dog densities. Further, this result is consistent with results reported in [17, Supplementary Material, p. 6), who find that reported bite incidence decreases with the urban proportion of population.

## PEP completions

The PEP Completions regression presented in Table [3,](#_bookmark31) Regression 3 in the main text represents a reduced form equation capturing drivers of both demand and supply of PEP. PEP Completions has a weak but significant positive relation to contemporaneous reported exposures. The fact that Reported Exposures is an order of magnitude higher than PEP Completions suggests that many cases are reported for which PEP is not indicated, and so the volume of reported exposures is apparently not a good indicator of aggregate PEP demand. More interestingly perhaps, PEP use is weakly negatively related to current Dog Rabies Cases (though also not statistically significant at conventional levels). One would expect that current Dog Rabies Cases would be positively associated with current PEP use if dogs are the culprit.

However, there is a relatively strong positive relationship between lagged canine rabies cases and current PEP completions — about a 1.3% increase per 10% increase in lagged dog rabies cases. PEP use is also positively related to lagged human rabies cases, with an estimated 2.9% increase in current PEP use in response to 10% more human rabies cases the previous year. Or inversely for our sample, as dog and human rabies cases decline, so does PEP use.

*Urban* and *Rural [human] population* have complicated relationships with PEP Completions, suggesting that urban populations have a lower propensity to PEP use conditional on rabies cases and exposures. This might make some sense if most exposures and rabies cases occur in rural areas and/or PEP access is weaker in urban areas.

Income and household costs of healthcare are strongly associated with PEP use. A 1% increase in Income is associated with a 2.3% increase in PEP Completions, conditional on other factors. PEP Completions is also positively associated with public per capita Health Expenditures, but negatively associated with per capita Out of Pocket health care expenditures. People may be more inclined to seek and receive PEP if their country’s health care infrastructure is effective, and if they have insurance or are subsidized such that their out-of-pocket expenses are lower.

## Human rabies cases

The incidence of Human Rabies Cases (deaths) is determined at the intersection of exposure and PEP completion, with a great deal of variation in the rate of infection given exposure. As discussed above, PEP use is an important endogenous factor affecting human rabies cases. PEP will be completed when risk is high given exposure, which in itself would lead to a positive correlation between PEP use and human rabies cases in aggregate data. At the same time PEP use itself presumably would reduce the incidence of death in the exposed population.

Based on the two-stage instrumental variable method described in Section S3, Table 3 Regression 4 in the main text shows that at 10% increase in PEP Completions decreases death by about 7.1%, all else constant. Without implementing the instrumental variable approach and using the original variable, this estimated impact is spuriously positive (0.42, p-value=0.016) as might be expected, since PEP is used when the likelihood of being infected is highest; this illustrates the need for an instrumental variables approach.

Conditional on PEP Completions, Human Rabies Cases is positively related to Dog Rabies Cases, with elasticity of 0.63. Thus, a 10% increase in Dog Rabies Cases is associated with about a 6.3% increase in Human Rabies Cases, holding PEP Completions constant. PEP completion numbers are about two orders of magnitude larger than the number of dog rabies cases in our sample, so a 1% increase in PEP Completions is much larger than a 1% increase in Dog Rabies Cases. These estimates and the weakly negative effect of Reported Exposures seem consistent with a scenario in which many of the human cases (deaths) in our data result from non-reported exposures.

Here results again suggest a negative relationship between Reported Exposures and Human Rabies Cases, though again it is not statistically significant at conventional test sizes (p-value=0.17). This negative effect could be a reflection of the impact of non-reporting of rabies exposures, and that if exposure reporting rates were higher, there would be fewer human death.

The indicator variable BMP takes the value 1 for Brazil, Mexico, and Peru, which are countries with larger rabies incidence at the beginning of the time series. Its inclusion is explained in the Methods section. It is strongly positively and significant, by design, to capture unobserved factors that set these three countries apart.

# S5. References for Supplementary Materials

1. Asamoah JKK, Oduro FT, Bonyah E, Seidu B. Modelling of Rabies Transmission Dynamics Using Optimal Control Analysis. Journal of Applied Mathematics. 2017. doi: 10.1155/2017/2451237
2. Fitzpatrick MC, Shah HA, Pandey A, Bilinski AM, Kakkar M, Clark AD, et al. One Health approach to cost-effective rabies control in India. Proc. Natl. Acad. Sci. U.S.A. 2016; 113: 14574-14581.
3. Hampson K, Dushoff J, Cleaveland S, Haydon DT, Kaare M, Packer C, et al. Transmission dynamics and prospects for the elimination of canine rabies. PLoS Biology. 2009; 7: e1000053.
4. Manski CF. The structure of random utility models. Theory and decision. 1977; 8: 229-254.
5. Brown BW, Walker MB. The random utility hypothesis and inference in demand systems. Econometrica. 1989; 57: 815-829.
6. Shim E, Hampson K, Cleaveland S, Galvani AP. Evaluating the cost-effectiveness of rabies post-exposure prophylaxis: a case study in Tanzania. Vaccine. 2009; 27: 7167-7172.
7. Cleaveland S, Fevre EM, Kaare M, Coleman PG. Estimating human rabies mortality in the United Republic of Tanzania from dog bite injuries. Bulletin of the World Health Organization. 2002; 80: 304-310.
8. Hampson K, Coudeville L, Lembo T, Sambo M, Kieffer A, Attlan M, et al. Estimating the global burden of endemic canine rabies. PLoS Negl Trop Dis2015; 9: e0003709.
9. Arellano M, Bond S. Some tests of specification for panel data: Monte Carlo evidence and an application to employment equations. Rev. Econ. Stud. 1991; 58: 277-297.
10. Arellano M, Bover O. Another look at the instrumental variable estimation of error-components models. J. Econom. 1995; 68: 29-51.
11. Blundell R, Bond S. Initial conditions and moment restrictions in dynamic panel data models. Journal of Econometrics. 1998; 87: 115-143.
12. Sargan JD. The estimation of economic relationships using instrumental variables. Econometrica: Journal of the Econometric Society. 1958; 26: p. 393-415.
13. Hansen LP. Large sample properties of generalized method of moments estimators. Econometrica: Journal of the Econometric Society. 1982; 50: 1029-1054.
14. Wilson P. The misuse of the Vuong test for non-nested models to test for zero-inflation. Economics Letters. 2015; 127: 51-53.
15. Cameron AC, Trivedi PK. Microeconometrics using Stata: Stata press College Station, TX; 2010.
16. Greene W. Econometric Analysis 6th Edition: Prentice Hall; 2008. pp. 683+.
17. WHO Rabies Modelling Consortium. 2019. The potential effect of improved provision of rabies post-exposure prophylaxis in Gavi-eligible countries: a modelling study. The Lancet Infectious Disease 19(1):102-111. <https://doi.org/10.1016/S1473-3099(18)30512-7>.
