# Supplementary material for: Healthcare demand in response to rabies elimination campaigns in Latin America
Source: PLoS Negl Trop Dis. 2019 Sep 26;13(9):e0007630. doi: 10.1371/journal.pntd.0007630 (PMC6762069; doi:10.1371/journal.pntd.0007630)
Supplement: S2 File — contains the submitted abstract translated into the Spanish language. (DOCX) [file pntd.0007630.s002.docx]

**Abstract (Spanish)**

La Organización Mundial de la Salud, la Organización Mundial de Sanidad Animal, y la Organización de las Naciones Unidas para la Alimentación y la Agricultura han declarado la meta de la eliminación por completo de las muertes humanas por rabia transmitida por mordidas de perros hasta el año 2030, y la Alianza Mundial para Vacunas e Inmunización ha incluido las vacunas antirrábicas humanas a sus proyectos para el periodo 2021-2025. Sin embargo, la eliminación rentable y sostenible requiere la comprensión de las complejas conexiones entre la vacunación antirrábica canina, la percepción humana del riesgo, y la toma de decisiones al respecto.

El objetivo de este artículo es cuantificar como la vacunación antirrábica canina afecta las muertes humanas por rabia, reportes de mordidas de perros, y uso de profilaxis antirrábica post-exposición (PEP por sus siglas en ingles). Para realizar esto, aplicamos análisis multivariante de regresión sobre cinco resultados relacionados a la rabia: (a) vacunación canina, (b) casos de rabia canina (c) reportes de personas expuestas (d) uso de PEP y (e) casos de rabia humana.

El análisis se basa en datos anuales agregados desde 1995 a 2005 en siete países de América Latina que tuvieron disminuciones drásticas de casos de rabia en caninos y humanos. Nosotros estimamos que (i) un aumento del 10% en vacunación antirrábica canina reduce casos caninos de rabia por 2.3%. (ii) Los reportes de personas expuestas disminuyen mientras los casos de rabia canina también de forma simultánea. Sin embargo, estas disminuciones son contrarrestadas por el aumento de personas expuestas (reportadas) por cada caso de rabia canina. Nuestro análisis parece indicar que este aumento es debido a una mayor percepción de riesgo por campañas antirrábicas. (iii) Un aumento del 10% en el uso de PEP disminuye muertes humanas por 7%, pero un aumento del 10% en vacunación antirrábica canina induce una disminución del uso de PEP de 2.8%. El efecto neto es que un aumento del 10% en vacunación antirrábica canina reduce muertes humanas en un 12.4%, aunque la efectividad marginal declina cuando también la incidencia de rabia canina declina. (iv) Aumentos de ingresos financieros y en gastos de salud pública a su vez incrementan la demanda de PEP. Los resultados recalcan la importancia de campañas masivas de vacunación antirrábica canina, mayor conciencia, acceso al tratamiento, y algoritmos clínicos para reducir los falsos negativos que llevan a la muerte tal como reducir falsos positivos que llevan a prescripciones costosas e innecesarias de PEP.
